# Exploring the experience of informal caregivers of dependent elderly people with the well-being valuation method

**DOI:** 10.3389/fpsyt.2025.1584632

**Published:** 2025-04-29

**Authors:** Tongzhou Lyu, Suwei Yuan, Yanping Xu, Wenwei Liu

**Affiliations:** ^1^ School of Politics and International Relations, East China Normal University, Shanghai, China; ^2^ Office of the Director, Ren Ji Hospital, Shanghai Jiao Tong University School of Medicine, Shanghai, China; ^3^ School of Social and Public Administration, East China University of Science and Technology, Shanghai, China; ^4^ College of Philosophy, Law and Political Science, Shanghai Normal University, Shanghai, China

**Keywords:** informal care, informal caregivers, subjective well-being, well-being valuation method, informal caregiver burden

## Abstract

**Introduction:**

Although informal caregivers are the primary providers of long-term care for the elderly globally, research on the impact of caregiving experiences on the well-being of caregivers is relatively limited.

**Methods:**

This study conducted interviews with 297 family informal caregivers in Shanghai, measured their subjective burdens, and applied the well-being valuation method to calculate the monetary value of the welfare changes resulting from caregiving burdens.

**Results:**

According to the findings, sub-dimensions of the Chinese version of the Caregiver Reaction Assessment (CRA) of disrupted schedule, lack of family support, and self-esteem were significantly related to caregivers’ well-being. For caregivers with average income and average CRA scores, the impact of caregiving experiences on their well-being level was equivalent to a monthly income reduction of 226.69 RMB (4.7% of their monthly income).

**Discussion:**

This paper provides a new and useful perspective for the evaluation of long-term care policies, and suggests prioritizing the alleviation of caregivers’ schedule and social relationship burdens, and understanding the ‘intrinsic coping mechanisms’ of caregiving responses from an economic perspective.

## 1 Introduction

Informal caregivers are non-professional persons (e.g., family members, friends, neighbors, etc.) who perform unpaid caregiving tasks (1, 2). The number of informal caregivers is quite substantial. One estimate from Europe suggests that the proportion of informal caregivers within the population ranges from 13% to 22% (3). Because of traditional family norms and a filial piety culture, most Chinese elderly people prefer home-based care (4, 5), and informal caregivers have always been the main providers of long-term care for frail family members in need (6).

Although unpaid, providing care is not without certain costs. Indeed, a wide range of research has studied the extensive negative impact of caregiving on the physical, mental, social, and economic experiences of informal caregivers. Systematic reviews show consistently lower perceived physical and psychological health conditions for informal caregivers compared to non-caregivers (7). Musculoskeletal discomfort (8, 9), depression (10–12), and anxiety (11, 13, 14) were found to be common among informal caregivers. A few investigations, however, examined the positive experiences of informal caregivers. For instance, feelings of higher intrapersonal satisfaction, demonstration of filial piety, and a better relationship with the care-receiver were determined to be contributors of a positive experience of informal caregiving in different research contexts (15–17). With the growing consensus on a holistic perspective of care experiences, instruments have been developed to measure both the negative and positive experiences of informal caregivers. These include the Caregiver Reaction Assessment (CRA) (18), the Carers of Older People in Europe (COPE) Index (19), and the Caregiver Strain Index Expanded (20).

To address caregiving burdens, supportive efforts, such as respite (21–23), skills training (22–24), and financial support (22, 23, 25), have been implemented in many countries and regions. Their effects were usually measured by comparing the targeted burdens/negative experiences pre- and post-interventions (26, 27). Shanghai is the ‘oldest’ city in China. In 2023, the population aged 60 and above in Shanghai reached 5.68 million, representing 37.4% of the total population (28). To promote the sustainability of family care, the government has implemented tax refunds, respite care, and publicly funded skill training programs for informal caregivers (29). However, the impact and effectiveness of these interventions have not been systematically evaluated.

Given the substantial hardships and challenges faced by informal caregivers, it is not surprising that they have been shown to have significantly lower levels of subjective well-being compared to non-caregivers worldwide (30–33). Regression analyses results indicated that informal caregivers’ well-being levels were associated with caregiving burdens, caregiving time, and other care-related factors, depending on the study sample characteristics, models adopted, and scales employed (34–36). In contrast, however, there is a general paucity of available literature on factors correlated with the improvement of caregivers’ well-being (37). Studies have employed various methods to quantify the economic value of objective caregiving burden, primarily in terms of caregiving hours. These methods include the opportunity cost method (38–40), the proxy good approach (38, 40, 41), contingent valuation (42–45), and the conjoint valuation method (46–48). Among these approaches, the well-being valuation method calculates the marginal impact of caregiving variables on well-being levels (49). This method allows for the quantification of both positive and negative caregiving experiences and their effects on overall well-being.

Overall, caregiver burdens and subjective well-being stressors have been investigated comprehensively. However, a few aspects remain to be adequately explored. First, current studies primarily focused on the various factors (predominately stressors) influencing the well-being levels of informal caregivers, but the extent of their impact has not yet been adequately quantified. Second, debate continues regarding how to best include caregivers’ experiences into economic assessments of informal carer support programs. The difficulties mainly comprise incorporating the comprehensive effects involved and integrating subjective perceptions into economic considerations. This study aims to elucidate the experience of informal caregivers in Shanghai, and to assess well-being losses and gains with the well-being valuation method.

## 2 Materials and methods

### 2.1 Sample and data collection

Informal caregivers were recruited by placing posters in communities and receiving recommendations from local social workers during June to December in 2019 in all nine subdistricts in Hong Kou District, Shanghai. Eligible participants were required to be informal caregivers of a dependent elderly adult aged 60 years or older in a home setting, spending at least one hour per day providing care. If an interviewee reported taking care of two or more elderly simultaneously, she/he was asked to indicate the one that she/he spent the most time taking care of. In addition, the informal caregivers should be at least 16-years-old and be able to understand and speak in Mandarin or Shanghainese. Finally, 297 informal caregivers were included in the study. Face-to-face interviews were conducted by five trained social workers. Each interview took 20 to 30 minutes to complete. The questionnaire was structured into three sections: socio-demographic information, life satisfaction, and caregiving-related questions. This arrangement aimed to minimize the potential for caregivers’ experiences to influence their assessment of life satisfaction.

### 2.2 Well-being valuation method

The well-being valuation method is one of the most recent approaches to monetizing the costs/benefits of non-market goods, e.g., environment (50–52), health problems (53–56), income inequities (57), etc. Several studies have also utilized the method to assign a monetary value to caregiving hours (58–61). This method suggests that well-being levels depend on various positive factors (e.g., her or his income), as well as negative factors (e.g., illness). The monetary equivalent costs of these associated factors can be calculated by the marginal rate of substitution of income and relevant covariates (62, 63). The econometric model can be illustrated as follows:


$$w_{i}=\;\alpha +\;\beta_{1}y_{i}+\;{\text{β}}_{2}{\text{C}}_{\text{i}}+{\text{β}}_{3}{\text{X}}_{\text{i}}+\;\epsilon_{i}$$

According to the model, the well-being level of caregiver i can be explained by her/his income $y_{i}$ , care-related experiences ${\text{C}}_{\text{i}}$ , and other demographic variates ${\text{X}}_{\text{i}}$ . α denotes the constant term; $\beta_{1}$ , ${\text{β}}_{2}$ , and ${\text{β}}_{3}$ are the regarding coefficient (vectors); and $\epsilon_{i}$ is the unobservable error term. Both income and care provision influence the respondent’s well-being level. The equivalent monetary costs of changes in care-related experiences can be estimated by using the ratio of the regression coefficients as follows:


$$\frac{\delta y_{i}}{\delta {\text{C}}_{\text{i}}}\;=\;-\;\frac{{\text{β}}_{2}}{\beta_{1}}$$

**Figure 1:**
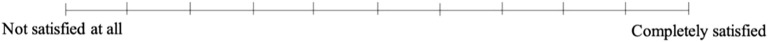
Happiness visual analogue scale (VAS).

Subjective well-being levels were measured by overall life satisfaction. To encourage the interviewees to incorporate all possibly influential factors into the evaluation, the happiness visual analogue scale (VAS) was included at the end of the questionnaire (**Figure 1**). The interviewed informal caregivers were asked to answer the following question, “All things considered, on a scale of 0 to 10, where 0 denotes ‘not satisfied at all’ and 10 denotes ‘completely satisfied’, how satisfied are you with your current life?” In the pilot study, a few respondents preferred to answer the VAS with their fingers using a touch screen. In this research, to enhance the accuracy of evaluation, the research team prepared both paper and electronic versions of the VAS to avoid measurement bias.

### 2.3 Variables and statistical method

#### 2.3.1 Caregiver experience assessment instrument

The Chinese version of the CRA was adopted as the caregiver experience assessment instrument (64). CRA measures both negative and positive experiences by five reaction dimension constructs (18). The reliability and validity of the instrument have been widely tested and confirmed in different cultures and caregiving situations (65–69). The CRA contains four subscales of stressors: “disrupted schedule”, “financial problems”, “lack of family support”, and “health problems”, and one subscale of positive reaction, “self-esteem”. There are 24 items, which include four inversely coded questions. To avoid respondents possibly confusing their reactions to caregiving with other experiences, CRA questions were posed immediately after items regarding caregiving. Moreover, the respondents were specifically reminded that answers to CRA-related questions should only be related to caregiving activities. Five Likert-type scales were employed, as the original scale suggested. The scores of each sub-scale were calculated by the arithmetic mean of the items.

#### 2.3.2 Demographic measures

Studies have reported that subjective well-being level is correlated with a number of socio-demographic characteristics (70, 71). The data of respondents’ gender, age, educational level, employment status, marital status, and health condition were collected. In addition, information regarding the respondents’ relationship to the dependent elderly and co-residence situation were captured. Educational level was categorized according to national education into 0–6 years, 7–15 years, and above 16 years. Health condition was collected through asking whether she/he was suffering from any chronic disease. To enhance the accuracy of income data, which is critical for the well-being valuation, the interviewed informal caregivers were asked to disclose their exact income amount in the previous month. The reported monthly income included all earnings in the form of money, salaries, pension, remuneration, profits from business(s), rent, financial benefits, gifts, etc.

#### 2.3.3 Analytic strategy

Numbers and percentages, means, and standard deviations were adopted in the descriptions of continuous and categorical data, respectively. Considering the diminishing marginal utility of income on well-being, the income amount was adapted into logarithm in analysis. Student-*t* test, one-way analysis of variance (ANOVA), and Pearson correlation were utilized to test the significancy of differences or correlations. Variables that were significantly associated with the dependent variable in the univariate analysis were included in the multivariate analysis. Ordinary least squares (OLS) regression analysis was employed to derive the coefficients for the well-being valuation calculation. The regression coefficients for each CRA subscale, along with the coefficients for the natural logarithm of income, were utilized to calculate the marginal rate of substitution between these two variables. The average income of the study sample served as a reference point for computing the monetary equivalent value. The significance level was set to 95%. Epidata version 3.2 and SPSS version 20.0 were used for data entry and statistical analysis, respectively.

## 3 Results

### 3.1 Descriptive analysis

Overall, the average life satisfaction score among the interviewed informal caregivers was 7.91 (SD = 1.27). No statistically significant differences in well-being levels were found based on gender (*p* = 0.878), age (*p* = 0.091), marital status (*p* = 0.067), relationship to the dependent elderly (*p* = 0.853), or co-residence (*p* = 0.818) (**Table 1**). However, respondents with higher educational levels (*p* < 0.001) and/or employment (*p* = 0.002) reported significantly greater well-being compared to other groups. Notably, a majority of the caregivers (67.0%) were suffering from at least one chronic disease, with healthier respondents exhibiting significantly higher life satisfaction (*p* < 0.001). Among the caregiving experience subscales, self-esteem received the highest reaction score, while financial difficulties had the lowest. All four subscales of the Caregiver Reaction Assessment (CRA) were significantly associated with life satisfaction: disrupted schedule (*p* < 0.001), lack of family support (*p* < 0.001), financial problems (*p* = 0.014), and self-esteem (*p* < 0.001).

**Table 1:** Descriptive analysis.

	N (%)/Mean ( ± SD)	Life satisfaction	*p*
Gender			
Female	165 (55.6%)	7.92	0.878
Male	132 (44.4%)	7.89	
Age	56.48 ( ± 11.67)	-0.101^*^	0.091
Income (yuan)	4860.70 (± 1704.39)	0.458^*^	<0.001
Education level	(Missing = 12)		
0~6 years	9 (3.2%)	7.41	0.001
7~15 years	190 (66.7%)	7.72	
16 years of above	86 (30.2%)	8.31	
Employment status			
Employed	165 (55.6%)	8.11	0.002
Retired	86 (29.0%)	7.80	
Unemployed	46 (15.5%)	7.38	
Marital status	(Missing = 10)		
Married	264 (92.0%)	7.96	0.067
Divorced	15 (5.2%)	7.63	
Others	8 (2.8%)	7.21	
Sufferer of chronic disease	(Missing = 18)		
No	94 (33.0%)	8.55	<0.001
Yes	191 (67.0%)	7.47	
Relationship to the care-receiver	(Missing = 4)		
Spouse	84 (28.7%)	7.88	0.853
Child	209 (71.3%)	7.91	
Co-residence	(Missing = 18)		
Yes	151 (54.1%)	7.88	0.818
No	128 (45.9%)	7.92	
CRA			
Disrupted schedule	2.69 (± 0.96)	-0.340^*^	<0.001
Lack of family support	2.62 (± 0.85)	-0.274^*^	<0.001
Health problems	2.65 (± 1.22)	-0.012^*^	0.839
Financial problems	2.15 (± 0.48)	-0.143^*^	0.014
Self-esteem	2.88 (± 0.63)	0.250^*^	<0.001

*Pearson correlation.

### 3.2 Factors associated with informal caregivers’ life satisfaction

Results of multivariate analysis (R^2^ = 0.369, adjusted R^2^ = 0.355, *p* < 0.001) are presented in **Table 2** Income, health condition, and three subscales of CRA were found to be consistently associated with the well-being levels of informal caregivers. Higher income (β coefficient = 1.200, 95% CI β coefficient = 0.830 - 1.570, *p <*0.001) and healthier status (β coefficient = 0.574, 95% CI β coefficient = 0.298 - 0.850, *p <*0.001) were correlated with higher levels of well-being. Controlling for other variables, higher reaction assessment scores of disrupted schedule (β coefficient = −0.258, 95% CI β coefficient =-0.407- -0.109, *p* = 0.001) and lack of family support (β coefficient = −0.199, 95% CI β coefficient =-0.354 - -0.044, *p* = 0.013) were negatively associated with life satisfaction levels. The positive reaction construct self-esteem (β coefficient = 0.368, 95% CI β coefficient = 0.143 – 0.593, *p* = 0.002) was positively correlated with respondents’ subjective well-being levels.

**Table 2:** Results of multivariate regression analysis.

	Beta	Std. error	95% CI β coefficient	*t*	*p*
Ln(income)	1.200	0.189	0.830 - 1.570	6.347	<0.001
Sufferer from chronic disease-No	0.574	0.141	0.298 - 0.850	4.083	<0.001
Disrupted schedule	-0.258	0.076	-0.407 - -0.109	-3.402	0.001
Lack of family support	-0.199	0.079	-0.354 - -0.044	-2.513	0.013
Self-esteem	0.368	0.115	0.143 – 0.593	3.204	0.002

### 3.3 Well-being valuation of caregivers’ reaction

The results of the well-being valuation are presented in **Table 3** The average income of 4860.70 yuan was chosen for calculation. For an informal caregiver who received an average income, when the reaction score of disrupted schedule increased from 1 to 2, the influence of this change to her/his well-being level was equal to a decrease of income of 1165.90 yuan (which accounts for 24.0% of the monthly income). With the increase of CRA scores of disrupted schedule from 2 to 3, 3 to 4, and 4 to 5, the monetary equivalent to the impact on well-being level was 551.65 yuan (11.3% of monthly income), 361.14 yuan (7.4%), and 268.41 yuan (5.5%), respectively. Similarly, increases in the “lack of family support” reaction score from 1 to 2, 2 to 3, 3 to 4, and 4 to 5 were associated with well-being losses equivalent to monthly income decreases of 876.76 yuan (18.0%), 420.21 yuan (8.6%), 276.25 yuan (5.7%), and 205.75 yuan (4.2%), respectively. The positive experience of caregiving contributed to the maintenance of well-being levels, as hypothesized. To maintain the same level of well-being level, if an informal caregiver’s self-esteem increased from 1 to 2, this was equivalent to an increase of monthly income of 1744.75 yuan, which accounted for 35.9% of the average monthly income of the respondents. In total, according to the results of the well-being valuation calculations, for informal caregivers who reported CRA scores of disrupted schedule, lack of family support, and self-esteem all to be 2, the overall influence of caregiving reaction to their well-being level was equal to losing 297.91 (6.1%) of the average monthly income.

**Table 3:** Monetary value estimation of caregivers’ reaction.

	Disrupted schedule		Lack of family support		Self-esteem		Total	
	Amount	% income	Amount	% income	Amount	% income	Amount	% income
1 to 2	-1165.90	24.0%	-876.76	18.0%	1744.75	-35.9%	-297.91	6.1%
2 to 3	-551.65	11.3%	-420.21	8.6%	805.48	-16.6%	-166.38	3.4%
3 to 4	-361.14	7.4%	-276.25	5.7%	523.16	-10.8%	-114.23	2.4%
4 to 5	-268.41	5.5%	-205.75	4.2%	387.31	-8.0%	-86.85	1.8%

At the same CRA level, which means that all three dimensions received the same score, the overall caregiving reaction’s impact on well-being was always negative. For an informal caregiver who reported the average scores of disrupted schedule (2.69) and lack of family support (2.62), the subjective well-being level was estimated as a reduction in monthly income of 1540.15 yuan and 1133.17 yuan, respectively. Meanwhile, for an average positive reaction of caregiving (2.88), its contribution to well-being was equal to obtaining 2446.63 yuan in monthly income. In summary, for a respondent earning the average monthly income and reporting average scores across the caregiving reaction constructs, the combined well-being loss due to caregiving was equivalent to a monthly income reduction of 226.69 yuan (4.7%).

## 4 Discussion

Our study provides preliminary empirical evidence of monetizing informal caregivers’ reaction to caregiving on subjective well-being level. Our research has several implications for exploring and explaining informal caregiving experiences. A higher reaction to disrupted schedule and lack of family support was significantly associated with lower subjective well-being level. Disrupted schedules stem simultaneously from the certainty and uncertainty of caregiving. From the aspect of certainty, most of informal caregivers claimed to struggle with normalizing the care-receiver’s daily routine of eating, dressing, bathing, etc. From the aspect of uncertainty, taking care of a dependent elderly inevitably involved dealing with a wide range of unexpected incidents (72). For instance, any acute onset of illness could lead to the need to re-organize care activities and the caregiver’s own needs. The results emphasized the importance of alleviating the stress of maintaining set schedules. Moreover, respite care is one of most commonly provided approaches for allowing caregivers to achieve an more optimal balance between caregiving and personal activities (21, 73). In addition, the statistical results are consistent with prior research, and indicate that the lack of family support constitutes a key adverse experience in caregiving activities. (34, 74). This experience may also be more pronounced in families influenced by traditional Chinese culture, leading to a greater impact on well-being levels. Studies have demonstrated that the utilization of electronic devices and online tools can efficaciously facilitate informal caregivers in acquiring social aid and resources (75). Besides, empirical evidence confirmed that the employment of electronic devices and the Internet can proficiently ameliorate the orchestration of schedules (76, 77). Therefore, this paper posits that electronic devices and internet-based support programs may potentially serve as promising remedies for mitigating these two substantial reactions to caregiving.

It is also important to note that the participants in our study exhibited considerable positive experiences from caregiving, and the reaction of increased self-esteem significantly enhanced the subjective well-being ratings. As mentioned above, while some literature has explored the positive experiences of caregiving to a certain extent, future research should place a greater focus on elucidating their impact on caregivers’ well-being and the precise mechanisms underlying these effects.

The results of the well-being valuation offer an illuminating explanation for the perseverance of informal caregivers in performing stressful tasks and the inherent coping mechanisms of individuals in caregiving roles, which might help to mitigate the influences of negative experiences. The findings indicated a limited net impact, i.e., even when the scores for the three caregiver reaction items were at their highest, their combined impact on caregivers’ overall well-being was equivalent to a reduction of 86.85 yuan in monthly income, which accounts for 1.8% of the average monthly income’s effect on total well-being.

The results of the well-being valuation method, however, should be interpreted with caution. Specifically, the net well-being loss only measures the extent of the caregivers’ overall reactions, but it should not be understood as a comprehensive reflection of all burden dimensions. Indeed, the mere presence of burdens does not dissipate due to the increase in welfare levels that they may concurrently bring, which can be understood from the venerated maxim, ‘A heart ailment requires a heart remedy.’ Although the burden that one feels in social relationships can be accurately assessed using the welfare valuation method, the provision of equivalent economic compensation will only improve their overall welfare level, without truly alleviating the burden from social relationships. Therefore, when considering relevant caregiver support policies and measures, it is essential to take the specific circumstances of each burden dimension into account in order to avoid addressing only superficial aspects of the problem.

Our results also offer several useful policy implications. First, application of the well-being valuation method to assessing caregivers’ well-being can serve as a potentially effective tool for cost-efficiency analysis of supportive programs aimed at improving caregivers’ well-being. By translating the subjective reactions of support into quantifiable metrics, various factors can be weighted and calculated for accurate and effective program appraisals. Second, the research team has previously applied this research method to determine the marginal effect of objective caregiving hours on well-being levels and has used it as a reference to estimate informal caregivers’ willingness to pay for formal care (61). As previously discussed, from a utilitarian perspective, supportive programs could easily overlook the complexity of caregiving experiences. It is crucial, for example, to acknowledge that equivalent financial support only restores an individual’s general well-being level but does not alleviate the negative experiences associated with caregiving.

This study possesses several limitations that are worth noting. First, due to research resource constraints, the sample size is limited to Shanghai, which may diminish its representativeness. Furthermore, given the significant social, cultural, and economic disparities between rural and urban areas in China, the findings may be subject to a certain degree of urban bias. Interested researchers may thus consider applying this method to a broader sample to obtain more generalized and robust conclusions. Second, due to the econometric requirements of the well-being valuation method and the exploratory nature of the study, this study employs the OLS regression method to ensure simplicity and clarity in the calculations. Since objective caregiving burdens—such as the intensity and duration of care, as well as the physical and dependency status of the care receiver—have been shown to impact subjective caregiving burdens in various research contexts (78–81), this study exclusively included subjective reactions to care in the statistical analysis to prevent collinearity and ensure estimation accuracy. Future research should consider adopting a broader range of econometric models that account for diversity and comprehensiveness, in addition to incorporating qualitative research methods, to better elucidate the underlying mechanisms.

## 5 Conclusions

Our study provides a preliminary exploration of informal carers’ experiences with the well-being valuation method. The equivalent amount of monetary value of caregivers’ reactions to caregiving were calculated and analyzed. The findings suggest prioritizing the alleviation of burdens related to informal caregivers’ disturbed schedules and family relationships. In addition, the importance of accurately understanding the “internal coping mechanisms” of informal caregiving reactions was emphasized. This study also provides a novel and useful perspective for economic evaluation of relevant caregiving supportive programs.

## Data Availability

The raw data supporting the conclusions of this article will be made available by the authors, without undue reservation.
